# Oscillation effect dataset on the measurement accuracy of load-cell sensor applied to the weigh basket

**DOI:** 10.1016/j.dib.2021.107453

**Published:** 2021-10-06

**Authors:** Nunik Destria Arianti, Muhamad Muslih, Agustami Sitorus, Ramayanty Bulan

**Affiliations:** a Department of Information System, Nusa Putra University, Sukabumi, Indonesia; b Research Center for Appropriate Technology, National Research and Innovation Agency (BRIN), Subang, Indonesia; c Department of Mechanical Engineering, Nusa Putra University, Sukabumi, Indonesia; d Department of Agriculture Engineering, Faculty of Agriculture, Syiah Kuala University, Banda Aceh, Indonesia

**Keywords:** Agricultural engineering, Precision for appropriate technology, Grains, Mass oscillation

## Abstract

The dataset in the form of weight, which will be closely related to the moisture content of agricultural products that have been dried in a mechanical dryer, is important to know precisely. Changes in these properties occur very quickly, so that it is important to prepare a system that is integrated with the mechanical dryer, especially the fluidized dryer type. On the one hand, the fluidizing dryer causes a shock to the weigh basket, connected to the weighing system mechanism. Therefore, this article collects a dataset of the weight of agricultural products (maize and soybeans) that have experienced shocks on two weigh baskets that could potentially be used in fluidization-type mechanical dryers. A load-cell sensor connected to a weigh basket is used to measure the weight of the agricultural product. A new generation of IoT techniques will control the sensor. Its microcontroller will send data to the cloud server via an internet network. There were a total of 120 treatments in the raw dataset. For agriculture engineering researchers, this data will provide benefits in measuring the weight of agricultural material in the form of grain dried in a mechanical dryer, especially the type of fluidized dryer, it can be more accurately explained.

## Specifications Table

**Table:** 

Subject	Engineering
Specific subject area	Agricultural engineering; weight of grain in shaken weigh baskets
Type of data	Graph
How data were acquired	The weight dataset for agricultural products is carried out in two weigh basket models. The grains in this sample include maize and soybeans. Five weight variations of each of these grains were investigated (100, 500, 1000, 1500, 2000 g). The amount of oscillation from each applied treatment was 30, 28, 26, 24, and 22 cm. A load-cell sensor is used to measure the change in weight of the grain during the shock treatment in the weigh basket. The sensor is controlled by an IoT-based microcontroller. Data will be sent automatically to cloud data servers via internet network data communication.
Data format	Raw
Parameters for data collection	The treatment parameters from the dataset in this article consist of the form weighing basket, the type of agricultural product in the form of grain, the grain's weight, and the shock as an oscillating effect. A total of 120 experiments have been mentioned in this article.
Description of data collection	This dataset contains information on shocks' effect on changes in weight readings of agricultural products in the form of grains in a weigh basket. This weight is measured with a load-cell sensor with a 0.05% error. There are two types of weighing baskets used, i.e., type 1 (cylinder-shaped with a diameter of 275 mm and a height of 285 mm) and type 2 (in the form of a cylinder with a cone-shaped base with a diameter of 170 mm and a height of 110 mm). Weight treatment variations for each tested grain consisted of 100, 500, 1000, 1500, and 2000 g. The shocks were applied at the oscillation levels of 30, 28, 26, 24, and 22 cm.
Data source location	Data were collected in the Research Center for Appropriate Technology, National Research and Innovation Agency (BRIN), and the Mechanical Engineering Laboratory, Nusa Putra University, Indonesia.
Data accessibility	Datasets in this article are available. The repository used Mendeley data pada link: https://data.mendeley.com/datasets/nnb24g7w39/3 or DOI: 10.17632/nnb24g7w39.3
Related research article	Non-invasive moisture content measurement system based on the ESP8266 microcontroller (Sitorus et al., 2020). https://doi.org/10.11591/eei.v9i3.2178

## Value of the Data

• The dataset provides information on the impact of shocks when measuring the weight of agriculture, particularly grain for the fluidized style dryer.
• This dataset can be used by agricultural researchers and engineers to predict the weight and grain that is being dried in a fluidized form dryer. Besides, those who focus on agriculture engineering can benefit from these data.
• In future studies, the data set will be used to model the agricultural material, particularly as a grain dried with dryers, especially fluidized type dryers, in order to predict the measurement error.

## 1 Data Description

The state of the art research has been conducted, in particular, using a fluidized mechanical dryer type on the non-destructive measurement of the moisture content of a dried product [1]. One of the physical characteristics can be determined by measuring the weight of the dried material in the dryer tube [2,3]. The problem is that the time it takes to stand still for the dried product and does not affect its weight calculation after fluidization occurs is also an issue. Therefore, this dataset provides data on the magnitude of changes in weight measurement due to the agricultural material oscillating in the weigh basket. In this data set, two types of agricultural materials were tested, namely maize and soybean. Each material was tested on two types of buckets, namely bucket type-1, and bucket type-2. Data sets based on material and bucket types are collected in the same MS Excel (.xlsx) extension formats. Each data set file has sheets and columns, as described in Table 1.

**Table 1:** Description of the raw data columns and sheet on data set obtained using maize samples on bucket type-1.

Sheet	Column number	Parameter	Type or Levels	Unit	Explanation
Maize_bucket type-1	1	Data to-	1–4050	Unitless	

	2.	Treatment - Treatment weight (B) - Treatment amplitudo (A)	2000, 1500, 1000, 500, 100 0, 22, 24, 26, 28, 30	g cm	Code “B” to show weight sample in bucket and code “A” to show amplitude applied to the bucket

	3	Initial massa	2000, 1500, 1000, 500, 100	g	

4	Measured grain weight	-	g	

5	Error	-	%	

Resume treatment	1	No	1 - 30	Unitless	Number of samples
	2	Code treatment	-	Unitless	

	3	Total	-	Unitless	Total data for each treatment

	4	Percentage	-	%	Percentage of data in each treatment

	5	No	1 - 30	Unitless	Number of samples have been ascending based on total data per treatment

6	Code treatment	-	Unitless	

7	Total	-	Unitless	

8	Percentage	-	%	

9	Commulative percentage	-	%	

Graph	Graph 1	-	-	-	To show the error rate of measuring the weight of maize in a shaken basket type-1

Graph 2	-	-	-	To show a Pareto chart for the error rate of measuring the weight of maize grain in a shaken basket type-1

**Fig. 1:**
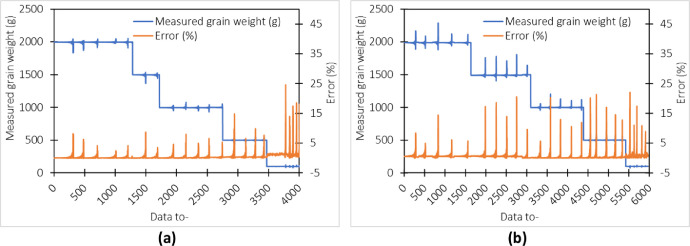
Error rate of measuring the weight of maize in a shaken basket (a) type-1 (b) type-2.

Fig. 1 indicates each weight treatment error for weighing baskets type-1 and type-2 on maize. It can be seen that every time a shock is made to the weigh basket, there is a change in the weight reading of the product a few times before returning to the previous weight. For basket type-1, the lowest weight read error, equal to 100 g, is observed. It is different from the type-2 weigh basket, where almost every mass has the same error. However, the highest error occurs at a treatment stage of oscillation of 30 cm.

**Fig. 2:**
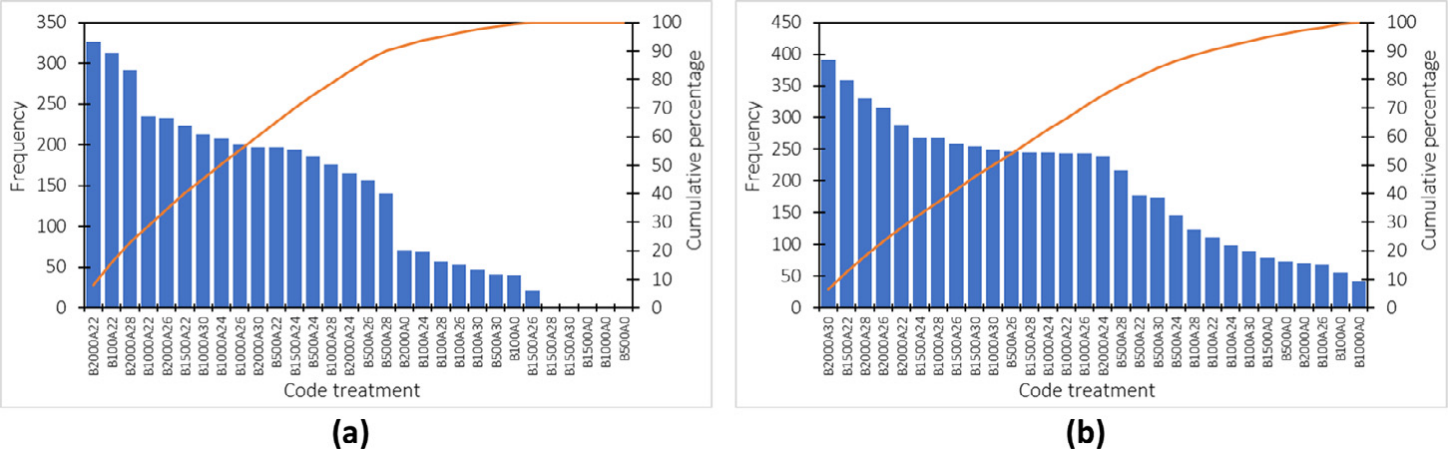
Pareto chart for the error rate of measuring the weight of maize grain in a shaken basket type-1 (b) type-2.

The pareto graph of the type of maize grain is presented in Fig. 2. The treatment of 2000 gr with a mass always provides the highest data frequency for both baskets tested types. The treatment codes in Figs. 2 and 4 consist of code “B” to represent weight and code “A” to represent amplitude. The number after the code “B” indicates the amount of weight applied to the test, and the number after the code “A” indicates the magnitude of the shock amplitude applied in the test.

**Fig. 3:**
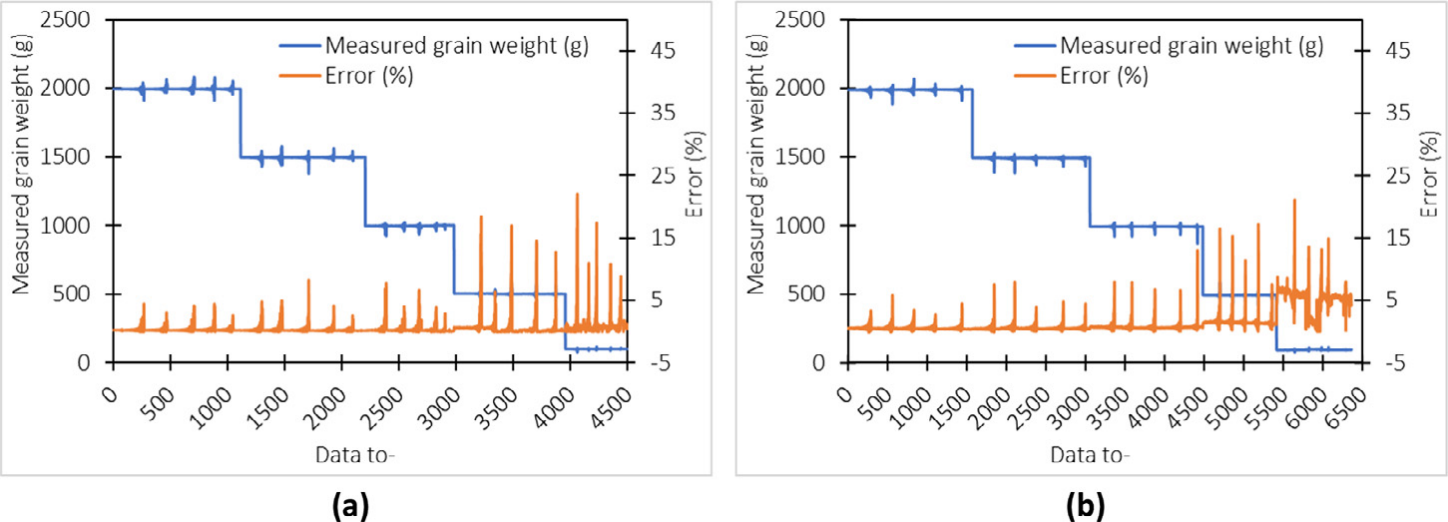
Error rate of measuring the weight of soybean grain in a shaken basket (a) type-1 (b) type-2.

The error of each weight treatment for weighing baskets type-1 and type-2 on soybean grains is presented in Fig. 3. It can be seen that every time a shock is made to the weigh basket, there is a change in the weight reading of the product a few times before returning to the previous weight. Like the type of maize, there is a more significant weight reading error at a lighter mass. This may be caused by the lighter the object being measured, the external factors such as environmental air will affect the sensor reading process which results in a larger reading error. For example, the lighter the object to be measured, the greater the environmental factors like wind, temperature, and relative humidity that interfere with the system's stability so that the error in the measurement results tends to be greater.

**Fig. 4:**
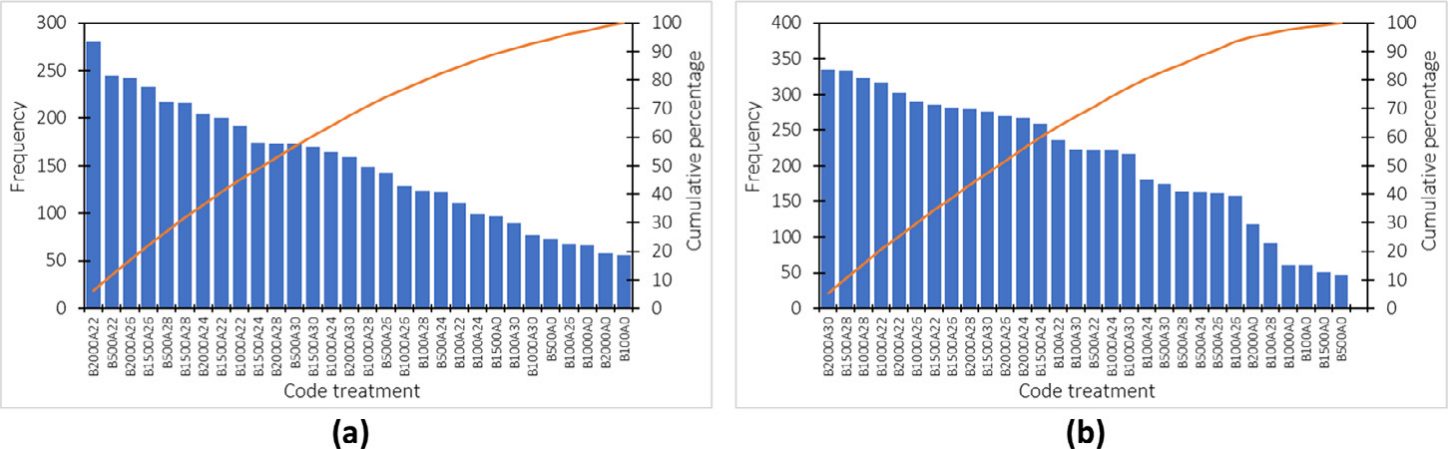
Pareto chart for the error rate of measuring the weight of soybean grain in a shaken basket (a) type-1 (b) type-2.

Fig. 4 shows the pareto graph of the soybean grain types. It can be seen that the treatment with a mass of 2000 gr always provides the most significant data frequency for the two types of baskets tested. This phenomenon is the same as in the previous experiment with maize grain. This may mean that the grain type with the same weight does not influence the weight measurement error.

## 2 Experimental Design, Materials and Methods

**Fig. 5:**
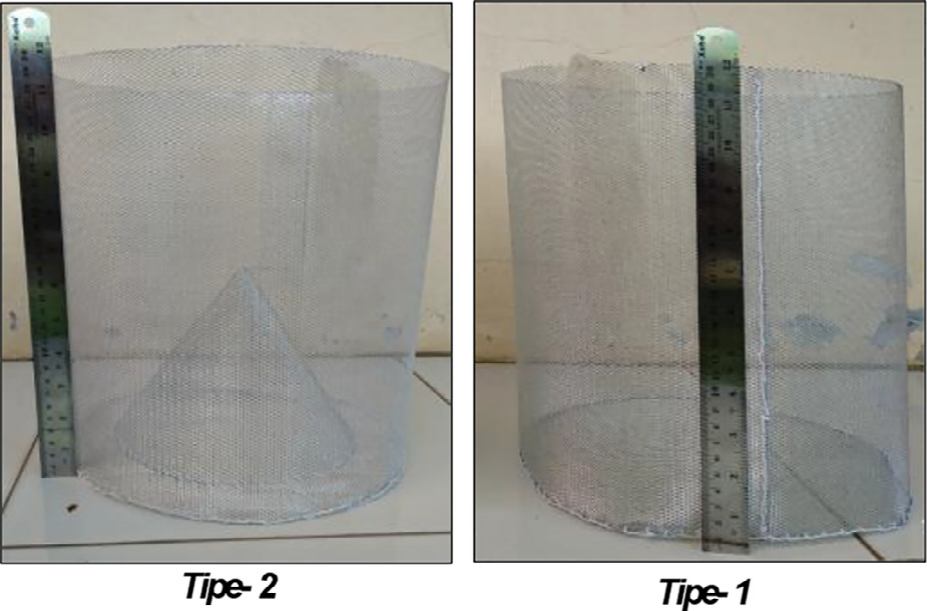
Types of weigh baskets.

Data acquisition used two types of weighing baskets, namely type-1 and type-2 (Fig. 5). Each basket is cylindrical with a diameter of 275 mm and a height of 285 mm. For type-1 weigh baskets, the bottom is only flat, and for type-2 baskets, the bottom is cone-shaped with a diameter of 170 mm and a height of 110 mm.

**Fig. 6:**
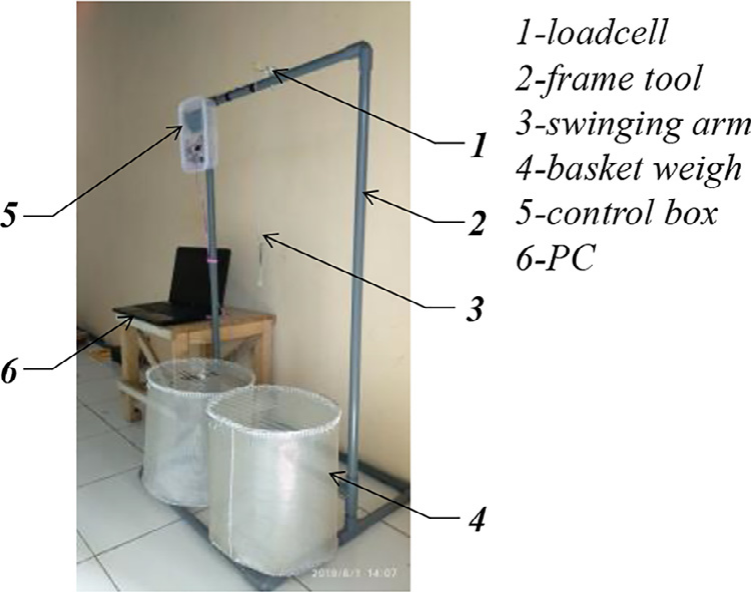
Apparatus experiment [1].

The grain weight oscillation dataset was acquired using the apparatus as shown in Fig. 6. In general, weight oscillation data is measured using a load-cell sensor connected to the weigh basket. The sensor is controlled by an IoT-based microcontroller. The measured data will be sent using the internet network to the cloud data server. Then the data can be accessed via a personal computer.

The collected dataset consisted of several types of treatment, namely the type of grains used, the type of weighing basket, the weight of the material fed, and the amount of oscillation applied. The types of grains used are agricultural products, namely maize, and soybeans. The types of baskets used are type-1 and type-2. Grain weight fed consists of 100, 500, 1000, 1500, and 2000 g. The oscillations applied were 30, 28, 26, 24, and 22 cm, and the treatment was without oscillation.

## Ethics Statement

No concern.

## CRediT authorship contribution statement

**Nunik Destria Arianti:** Investigation, Resources, Writing – review & editing, Funding acquisition. **Muhamad Muslih:** Investigation, Resources, Writing – review & editing, Funding acquisition. **Agustami Sitorus:** Conceptualization, Methodology, Software, Validation, Formal analysis, Data curation, Writing – original draft, Writing – review & editing. **Ramayanty Bulan:** Data curation, Resources, Supervision, Writing – review & editing.

## Declaration of Competing Interest

The authors declare that they have no known competing financial interests or personal relationships which have, or could be perceived to have, influenced the work reported in this article.
